# A Bayesian approach to modelling the impact of hydrodynamic shear stress on biofilm deformation

**DOI:** 10.1371/journal.pone.0195484

**Published:** 2018-04-12

**Authors:** Oluwole K. Oyebamiji, Darren J. Wilkinson, Pahala Gedara Jayathilake, Steve P. Rushton, Ben Bridgens, Bowen Li, Paolo Zuliani

**Affiliations:** 1 School of Mathematics, Statistics and Physics, Newcastle University, Newcastle upon Tyne, NE1 7RU, United Kingdom; 2 School of Engineering, Newcastle University, Newcastle upon Tyne, NE1 7RU, United Kingdom; 3 School of Natural and Environmental Sciences, Newcastle University, Newcastle upon Tyne, NE1 7RU, United Kingdom; 4 School of Computing Science, Newcastle University, Newcastle upon Tyne, NE4 5TG, United Kingdom; University of Notre Dame, UNITED STATES

## Abstract

We investigate the feasibility of using a surrogate-based method to emulate the deformation and detachment behaviour of a biofilm in response to hydrodynamic shear stress. The influence of shear force, growth rate and viscoelastic parameters on the patterns of growth, structure and resulting shape of microbial biofilms was examined. We develop a statistical modelling approach to this problem, using combination of Bayesian Poisson regression and dynamic linear models for the emulation. We observe that the hydrodynamic shear force affects biofilm deformation in line with some literature. Sensitivity results also showed that the expected number of shear events, shear flow, yield coefficient for heterotrophic bacteria and extracellular polymeric substance (EPS) stiffness per unit EPS mass are the four principal mechanisms governing the bacteria detachment in this study. The sensitivity of the model parameters is temporally dynamic, emphasising the significance of conducting the sensitivity analysis across multiple time points. The surrogate models are shown to perform well, and produced ≈ 480 fold increase in computational efficiency. We conclude that a surrogate-based approach is effective, and resulting biofilm structure is determined primarily by a balance between bacteria growth, viscoelastic parameters and applied shear stress.

## Introduction

Water is crucial for life on earth and is valuable also for its supporting role in ecosystem function. With growing global population and climate change induced water crises, there is an increase in the amount of wastewater for municipal uses and this might lead to a shortage of drinkable water. Biofilm technology is being deployed in the management and treatment of wastewater. A model is required that describes the individual processes in the wastewater treatment system. The simulation of microbial communities has important application in wastewater treatment studies. Wastewater treatment plants are open systems that depend on many species of bacteria to form a microbial community for the transformation of waste into biomass and other substances. According to [1], biofilms are regarded as the commonest form of bacteria on earth.

It has been established that the growth, structure and performance of bacteria biofilms are strongly affected by the hydrodynamic shear force. It is increasingly being recognised that hydrodynamic shear stress has a significant role to play on the deformation of biofilms and detachment of bacteria. There has been a large number of research projects dealing with the assessment of the impact of hydrodynamic stress on biofilms deformation. [2] observed that steady state structures of biofilms are strongly affected by the hydrodynamic shear stress. The general understanding of the influence of bacteria detachment is documented in [3–5].

The detachment process is an essential mechanism for removal of biomass from biofilm thereby controlling the biofilm key processes like growth, development and performance [2, 6, 7]. Moreover, [8] and [4] identified five different mechanisms of biomass detachment in biofilm while recently [3] focus on just only three out of these processes. The first category is the shear detachment which occurs as a result of fluid flow in the bacteria compartment, and an erosion detachment which is breakage of small particles from the surface of biofilm into bulk fluid. The third type is the nutrient-limited detachment which is associated with insufficient nutrient effects. However, [6] and [9] limit their attention to erosion (small-particle loss) and sloughing detachment of relatively large portions of the biofilm. In a similar vein, [9] noted that the biofilm simulation subjected to an erosion type of detachment event has the potential for making the biofilm surface smoother while sloughing type detachment can cause an increase biofilm surface roughness. In other words, the detachment phenomenon is a significant determinant of the shape, composition and structure of emerging biofilm.

We know that biofilm growth simulation is computationally demanding, and most of the available studies on biomass detachment usually base their inference on a relatively small sample of simulation data. For instance, [3] consider only three parameter values for shear, nutrient-limited and erosion detachment coefficients in their experiments while the consideration of only six parameter values is used as detachment parameters in [5]. The limitation of these studies is their lack of sufficient data to make a rigorous validation for testing how shear forces influence bacteria detachment. Similarly, [5] and [3] use only the simple detachment rate and probabilities in their studies but fail to incorporate the knowledge of mechanical interactions among the particles in their simulations, while [10] and [11] completely neglect the effect of biomass deformation in their studies.

The simulation of the impacts of shear flow on the size and structure of biofilm is undertaken in this paper such that the shear force from the fluid flow on mature biofilm leads to deformation and eventual breakage as an emergent event. The approach is computationally demanding as noted in some literature eg [5] because of the partial differential equation (PDE) necessary to model the mechanical forces in the system.

To gain deeper insights into how the shear force affects the deformation of biofilm, we develop a novel surrogate-based technique for solving this problem. A surrogate model is a simplified statistical approximation to an expensive computer model, often referred to as a “statistical emulator” [12] for predicting the shearing behaviour of model systems without having to rely on an expensive simulator. We are interested in strengthening the study of the essential role of shear stress breakage of microbial aggregates. We note that the crude parameter scans used in [3, 5] is not an ideal approach to fully understand the emerging properties of the microbial organism, and could be improved using a properly designed experiment. Our approach is to use advanced statistical techniques based on a large ensemble of simulation data to make a rigorously tested and validated assessment of the effect of shear force on microbial deformation. This approach will provide new insights into how quantitative statistical techniques can be used to simplify and study this complex problem.

The simulation data we analysed in this paper are from an expensive dynamic model. There have been a large number of studies that have examined data from a dynamic simulator. For instance, [13] emulated the characterized biofilms and floc outputs of an individual-based (IB) model of microbial communities using statistical principles of dynamic emulation while [14] focus on a low-order dynamic model that approximates the response of the high-order dynamic simulator at a low computational cost. [15] described a Bayesian method for quantification of uncertainty in complex computer models while [16] applied a Bayesian technique to calibrate computer models. Also, [17], focusing more on heterogeneous data, used a hybrid Markov chain Monte-Carlo method.

Bayesian computations have extended and broadened the scope of statistical models that can be handled in practice due to the development of Markov chain Monte Carlo (MCMC). However, if the model errors have a Gaussian distribution and a given form of the prior distribution is assumed, then the posterior distributions of the model’s parameters can sometimes be obtained analytically, without MCMC. A major benefit of using Bayesian regression is the provision of a measure of uncertainty in its analysis. The disadvantage is that, computationally, it can be very demanding. This is not a serious drawback for the problems we addressed in this paper because the sample size is moderate and the simplifying assumption we made under the DLM implementation also reduces the computational expense.

The two outputs we consider to model in this paper are the expected number of detachment events (count data) and volume of detached clusters. The traditional approach for modelling event-count data is to use a Poisson model. In the earlier studies of [18], a Bayesian Poisson regression model based on the Gibbs sampler was used while [19, 20] apply a similar Bayesian Poisson regression for modelling the crowd counting and injury count data respectively. We use a 3D individual-based model simulation of microbial organisms incorporating a fluid flow that is based on LAMMPS (Large-scale Atomic/Molecular Massively Parallel Simulator), a classical dynamical model for particle simulation. This simulator was enhanced to incorporate biological and physical processes to model bacterial growth, decay and mechanical interactions among bacteria cells [21].

We know from the available literature that the impact of hydrodynamic shearing force on the biofilm fragmentation has not been thoroughly studied using quantitative statistical techniques. The primary objective of this work is to investigate the effect of shearing force on the biofilm deformation and bacteria detachment using a surrogate-based method. Firstly, we assume that the biofilm fragmentation occurs as an event and we proceed by examining the extent to which shearing force impacts on the hazard of a bacteria detached from a parent biofilm.

Secondly, we quantify the relationship between the average number of shearing events per unit time and some covariates like total number of particles, shear rates, biofilm height, mass and extracellular polymeric substance (EPS) composition, using a novel combination of Bayesian Poisson regression and dynamic linear models to study the biofilm detachment problem. Bacteria are embedded in a sticky extracellular polymeric substance (EPS) produced by bacteria themselves and mostly composed of polysaccharide, proteins and nucleic acids etc. EPS helps for the structural integrity of the biofilm and hence biofilms can resist applied external forces. It also plays a significant role in microbial competitions in a multi-species biofilm [21–23].

We then predict the total volume of detached clusters per unit time as a continuous function of the predicted number of shearing events, shear rates and other covariates given in Table 1 using a dynamic linear model (DLM). This modular approach will enable us to predict the distribution of detached clusters over time. We describe the models and simulation data utilised for the analysis in Section 2. In Section 3, we describe the Bayesian methods including the dynamic linear models and Poisson regression. Section 4 provides the results of the analysis including the sensitivity analysis. Section 5 presents the discussion and concluding comments.

**Table 1:** IB model parameters.

Index	Parameters	Symbol	Values	Units	References
1	Substrate affinity	*K*_*s*,*HET*_	0.000035	kg*m*^−3^	[26]
2	Max. specific growth rate	*μ*_*m*,*HET*_	1.000000	*h*^−1^	[27]
3	Yield coefficient	*Y*_*HET*_	0.610000	$\frac{gCOD}{gCOD}$	[28]
4	Shear rates	*γ*	0.250000	s^−1^	chosen
5	Spring coef for collision	*K*_*n*_	1 × 10^−4^	*Nm*^−1^	[29]
6	Viscous coef for collision	*γ*_*n*_	1 × 10^−5^	*s*^−1^	Chosen
7	EPS stiffness	*K*_*e*_	5 × 10^9^	*s*^−2^	[30]
	Dimension	*L*_*x*_ × *L*_*y*_ × *L*_*z*_	200 × 40 × 100	*μm*^3^	-
	Cartesian grid cells	*N*_*x*_ × *N*_*y*_ × *N*_*z*_	30 × 12 × 30	-	

## Materials and models

### Simulation model

The present study models the biofilm that might be found in a wastewater treatment plant (WWTP) at the individual microbe level since pilot scale plants and laboratory scale experiments of wastewater treatment plants WWTP are expensive, cumbersome, non-invasive and often cannot provide information at the micro-scale, which is required for operational optimisation of WWTP. The mathematical models used for biological treatment can be mainly divided into two general classes according to the way the biomass is represented: continuous and discrete models. In the present work, a discrete individual-based (IB) model is used. Biofilms are the aggregated microbial communities attached to surfaces. We have one functional group of microorganisms and one dormant state as soft agents within the present model. The microorganisms are heterotrophs (HET) which consume organic carbon source and oxygen. The inert state is extracellular polymeric substance (EPS), secreted by some heterotrophs. In this agent-based model the EPS is modelled as discrete particles. The adhesive forces between EPS-EPS and EPS-Bacteria particles are modelled by springs in which the spring coefficient is a function of local EPS mass.

The dead agents are represented by soft spheres (labelled DEAD). Agents have four state variables: position, mass, radius, and type. This model consists of two principal submodels; one deals with the growth and behaviour of individual bacteria as autonomous agents (i.e., biological processes); the other deals with the substrate and product diffusion and reaction and fluid flow (i.e., physical processes). Each cell grows by consuming the substrate and divides when a certain mass is reached. When agents grow and split, the system deviates from its mechanical equilibrium due to some extra pressure built-up in the biomass.

Depending on the net force acting on each agent, resulting from its spatial interaction with other local agents, the position of each agent is updated using the Discrete Element Method (DEM). In DEM, contact, EPS adhesion and shear force are considered, and the position of agents are updated by solving Newton’s second law equation. For the substrates, Chemical Oxygen Demand (COD) is considered. The diffusion-reaction equation governs the substrate concentrations, and this transport equation is solved in a fixed Cartesian grid using a Finite Difference Method. This Newcastle University Frontiers in Engineering Biology (NUFEB) model extends the traditional IB model by incorporating mechanical interactions among bacteria. The NUFEB model is implemented in LAMMPS, an open-source *C*^+ +^ molecular dynamics code (http://lammps.sandia.gov/) [24]. More details about the model can be found in [21].

### NUFEB simulation data

The IB model was run with a series of growth parameters and shearing forces over an extended period using a Latin hypercube design to generate biofilms of various size. The LHD technique provides good coverage of the input space with a relatively small number of design points [25]. The parameters are varied within the range of ±50% of the standard values given in Table 1 for 140 training points and five replicates at each design point, due to the expense of this computer model. The parameters are *μ*_*m*,*HET*_ which is the maximum specific growth rate for HET, *K*_*s*,*HET*_ is the substrate affinity for HET. The parameter *K*_*s*,*HET*_ is an inverse measure of bacterium affinity to the organic substrate ie 1/*K*_*s*, *HET*_ can be considered as a representation of how easily nutrients are transported across a bacterial membrane. Other parameters are *Y*_*HET*_ which is the yield coefficient for HET growth, *γ* is the hydrodynamic shear rate, the spring coefficient for collision is denoted as *K*_*n*_, viscous coefficient for collision *γ*_*n*_ and EPS stiffness per unit EPS mass is denoted as *K*_*e*_; see Table 1 below, other simulation parameters are held constant; see Table A in S1 Text.

The design matrix is denoted as $X_{140\times 7}=\left(\theta_{p}^{i},p=1,\ldots ,7;i=1,\ldots ,140\right)$; where the subscript *p* represents the 7 model parameters that are varied in Table 1. The superscript *i* denotes the 140 different realisations. The simulator was run for 40000 s to grow the biofilm to a certain height without flow and then subjected the resulting biofilms to shear flow for an additional 200000 s where the biofilm detachment events occur. For each *i*, the output data is recorded at every 2000 s giving 120 time slices, *t* = 1, …, 120. The number of particles and volume of detached clusters lost from the parent biofilm was recorded for different shearing forces. We compute the biofilm height as given below. Other morphological characteristics like biofilm mass, total number of particles and EPS composition are also calculated for predicting expected number of shearing events over time.

The simulation box has dimension {0, *L*_*x*_} × {0, *L*_*y*_} × {0, *L*_*z*_}, where *L*_*x*_ = 200*μm*, *L*_*y*_ = 40*μm* and *L*_*z*_ = 100*μm*. To compute average biofilm height, the biofilms are partitioned into several smaller blocks or grids. The number of blocks in each direction are given as *N*_*x*_ = 30, *N*_*y*_ = 12 and *N*_*z*_ = 30. We compute the Euclidean distances between the center of each particle and the lattice blocks along the baseline (plane *z* = 0) to identify the occupied blocks. We, therefore, marked as “occupied” every block with one or more particle centers contained within it while the others are marked as “vacant”. The height *h*_*t*_(*x*, *y*) of the biofilm above each base block is defined as the maximum of the particle *z*− values of the occupied blocks. The biofilm mean height at time *t* is then given as $\bar{h}\left(t\right)=\frac{1}{L_{x}L_{y}}\int \int h_{t}\left(x,y\right)dxdy.$

### The scope of the problem

The problem we are addressing in this paper is the simulation of biofilm under shear stress, where the fluid force of appropriate magnitude flow on individual biofilms leads to the weakening of biofilms. This can result in eventual deformation and bacteria loss from the surface. Our focus in this paper is to be able to treat each shear phenomenon as an event, and we are interested in testing the feasibility of using a surrogate-based model for predicting expected number of shear events and size of detached clusters.

**Fig 1:**
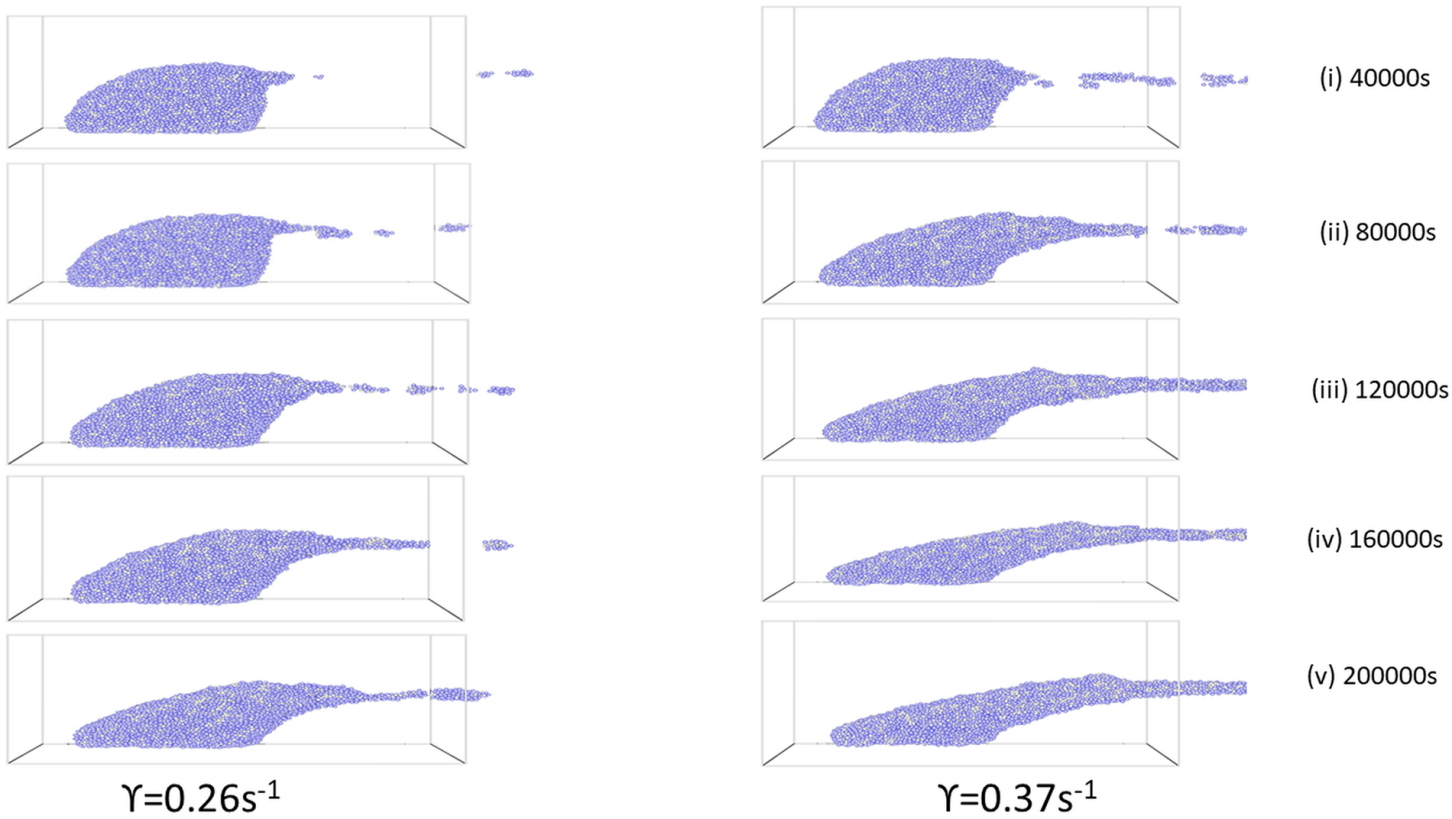
Biofilm structures showing temporal evolution of effect of shear rate on biofilm deformation and bacteria detachment for different shear rates for the period the flow is applied. Left-plot: *γ* = 0.26*s*^−1^ and right-plot: *γ* = 0.37*s*^−1^ for 40,000, 80,000, 120,000, 160,000 and 200,000 seconds respectively.

The knowledge about the emerging composition and structure of biofilms subjected to shear flow is useful to improve the performance and stability of wastewater reactors. Fig 1 represents a typical simulation of biofilm under two different shear rates at *γ* = 0.26*s*^−1^ and 0.37*s*^−1^ respectively, and we can see diverging temporal behaviours and structures when the shear flow is applied on a mature biofilm, the influence of cell detachment from the surface begins to emerge. The detachment phenomenon occurs when cohesive failures happen due to hydrodynamic shear force. At 40,000 s, the frequency of detachment events is higher for 0.37*s*^−1^ rate than for 0.26*s*^−1^. The detached clusters are moving out in the opposite direction to the bulk flow (shear flow from left-hand) as expected. The two shear rates give rise to different detachment patterns. We also see a gradual decreasing and flattening of the biofilms over time. In particular, the elongated filamentous cell clusters (streamers and clumps of cells) at a later time is obvious.

**Fig 2:**
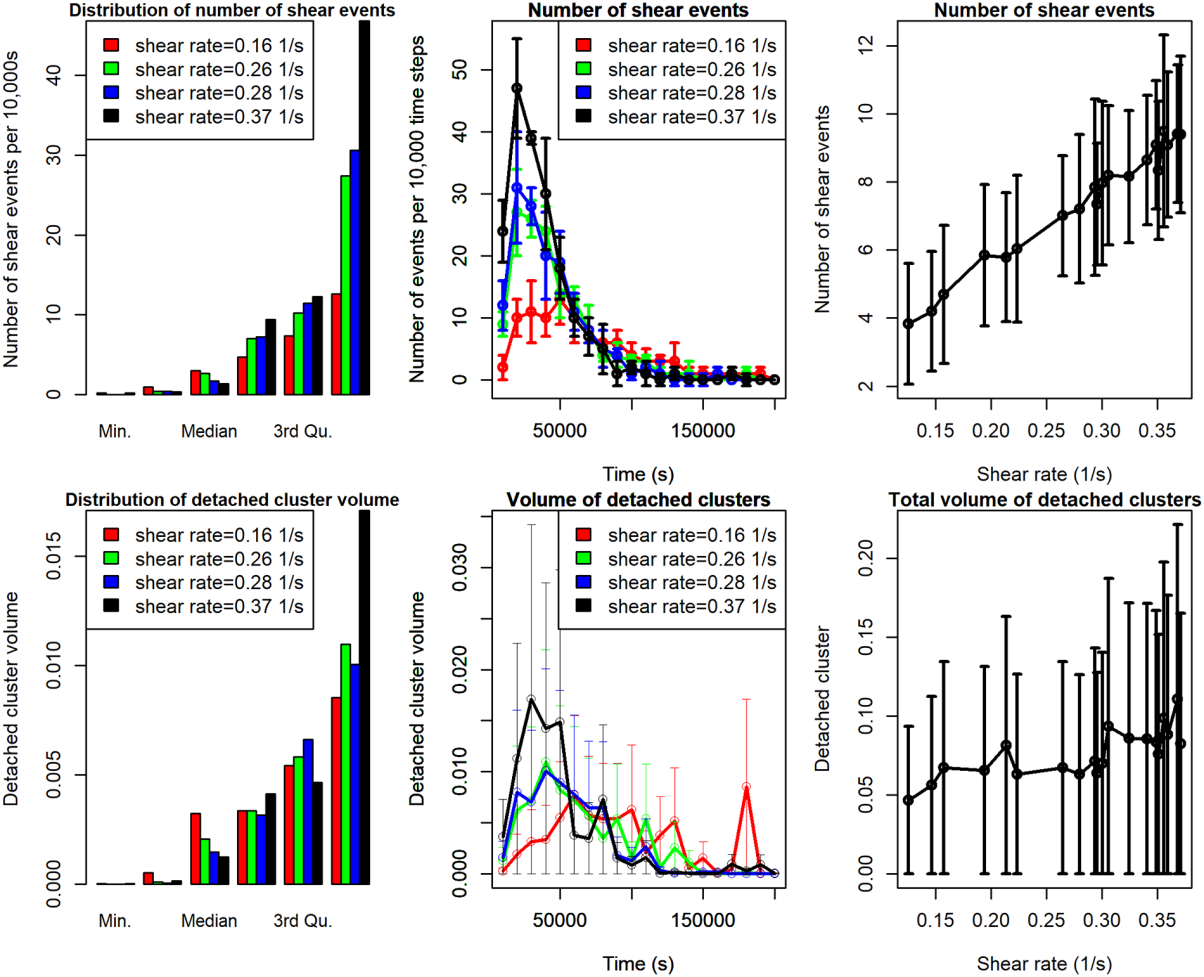
Density and time series plots for expected number of shear events and volume of detached clusters for different shear rates. The error bars show ±1 standard deviation calculated from five replicates. Note: volumes are normalized by their initial biofilm volume.

The density plots for the two outputs we are considering are given in Fig 2 (column 1). This enables us to look closely at the distribution of the two outputs to be analysed. It is apparent that each density plot has relatively different distribution under different shear rates and are highly skewed and nonnormal. The number of shear events is modelled using a Poisson distributed random variable because they are count data. We modelled the expected number of shear events function using a Poisson regression having an exponential-mean parameter that is a quadratic function of the explanatory variables. The inputs to the Poisson model are six variables namely biofilm height, mass, EPS composition, number of particles, shear rates and time. It is evident that density of detached clusters is left-skewed therefore logarithm transforming of these variables will reduce their skewness and make the data more interpretable to meet our dynamic linear model assumptions.

The corresponding time-series plots are displayed in Fig 2 (middle column) for different shear rates. There is a reduction in the number of shear events after reaching the maximum values for shear rates 0.37*s*^−1^, 0.28*s*^−1^ and 0.26*s*^−1^ respectively. A phenomenon which can be attributed to a more frequent detachment of smaller cells from biofilm surface at the beginning of experiment < 30,000*s*, often called erosion detachment. At a low shear rate of 0.16*s*^−1^, the number of shear events increases slowly and is relatively constant over time afterwards. We can conclude that the number of shear events under different shear rates has slightly varying patterns.

Similar to the number of events, the detached volume has a different trend under different shear rates which increase slowly over the time. For instance, at shear rates of 0.37*s*^−1^ and 0.28*s*^−1^ in Fig 2(middle column, bottom plot), there is a gradual increment in the volume of detached clusters due to top surface cells sheared off quite early after which there is a reduction and relatively constant detachment. This trend could be attributed to the particle at the top surface growing to a larger size because of better access to nutrients from the bulk medium. The effect of stochastic variation is pronounced because of large standard deviations around each output.

Fig 2 (column 3) shows the expected number of shear events averaged over all time and total volume of the detached cluster over all time. The growth in the number of events at higher shear rates agrees with the works of [31] and [32] who observed that doubling the shear stress frequency from 21.8 to 43.6mPa resulted in a multiple fold increase in detachment rate for both erosion and sloughing detachments.

It is apparent that while shear events increase linearly with an increase in shear rates, the total volume of detached clusters also increases nonlinearly with large stochastic variations around the mean values. We do not observe a reduction as reported in some literature, eg [32] recorded that there will be a decreasing in mean size of eroded clumps as shear rate increases.

To test the the influence of viscoelastic parameters, additional plots are given in the Supporting information (S4 Fig) where the time series of expected number of shear events and volume of detached clusters is examined for four different values of spring coefficients (*K*_*n*_) (0.0000503*Nm*^−1^, 0.0000831*Nm*^−1^, 0.0001387*Nm*^−1^, 0.0001501*Nm*^−1^). Similar to what we observe under the shear rates, higher spring coefficients gives larger number of shear events and volume of detached clusters for period < 50,000*s*. This pattern is expected because the repulsive forces increases as the spring coefficients increase making the particle to detach easily from the biofilm surface.

**Fig 3:**
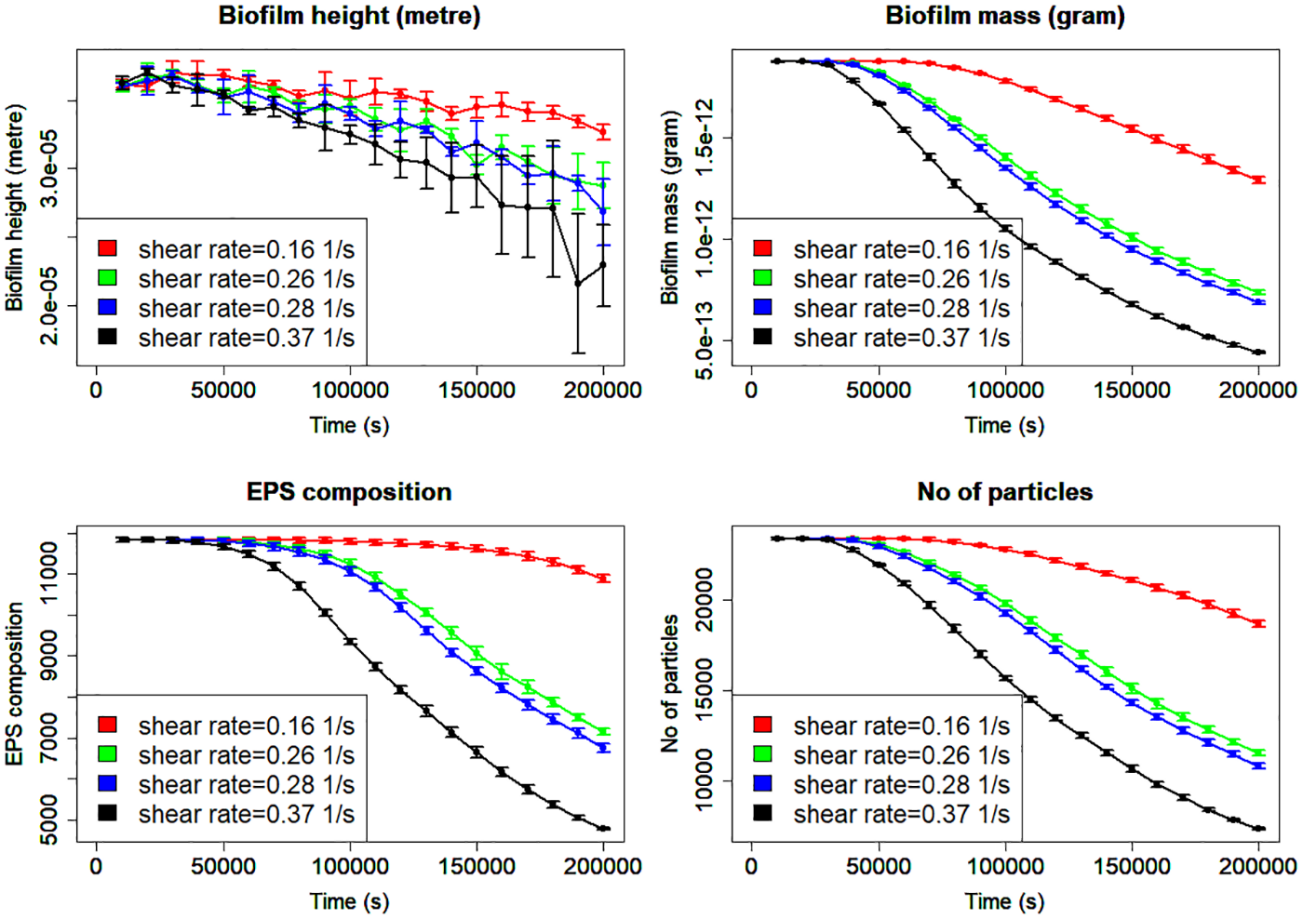
Dynamics of characterized outputs under different shear rates for biofilm height, mass, EPS composition and total number of particles at every 10,000s respectively. The error bars show ±1 standard deviation calculated from five replicates. These summary outputs are used as explanatory variables for predicting the expected number of shear events using Poisson regression. Note: EPS composition is the number of EPS particles.

Other summary outputs for the simulation data which are used as explanatory variables in the Bayesian Poisson modelling are displayed in Fig 3. There is a general decreasing trend over the time, those with higher shear rates (eg 0.37*s*^−1^) decline more rapidly than those with lower shear rate (eg 0.16*s*^−1^). While the biofilm height increases slowly between the first 30,000s, the EPS composition, biofilm mass and total number of particle plots are relatively constant within this corresponding period before a gradual decrease.

The EPS composition denotes the number of EPS particles in the simulation. EPS is a gel-like material that keeps bacteria together in the biofilms. Therefore high EPS composition rate will favour attachment of bacteria. EPS composition declines at a faster rate than biofilm height. The biofilm mass, EPS composition and total number of particles have a relatively similar trend which declines rapidly as expected because the clusters are being continuously detached from the surface. [33] also observed an exponential and asymptotic decrease of the biofilm thickness and mass when exposed to high shear stress. The effect of stochastic variation is considerably larger for biofilm height at the shear rate of 0.37*s*^−1^ and increases with time.

## Methods

The Bayesian framework involves combining observed data with a likelihood, and a prior distribution on unknown parameters to obtain the posterior distribution of the parameter given the data. It is often difficult to derive the posterior distribution in a closed form in most applications, and this often results in the use of Markov chain Monte Carlo simulation methods as an alternative. Markov chain Monte Carlo has been widely used in many complex applications for parameter estimation. [34] describes MCMC as a general tool for simulation of complex stochastic processes useful for making statistical inference. MCMC produces a sequence of random variables which can be used to approximate the true posterior distribution.

Gibbs sampling, for instance, is based on the principle that the knowledge of the conditional distributions is often sufficient to determine a joint distribution [35]. On the other hand, the Metropolis-Hastings algorithm allows one to make random draws from such a non-standard posterior distribution using proposal distributions. Moreover, [35] gives a detailed explanation of the theory behind Gibbs sampling while [36] highlights the theoretical background behind the Metropolis-Hastings algorithm.

### Dynamic linear models (DLMs)

Let us consider bacterial biofilms transported in a fluid flow with the following morphological characteristics measured on them. The volume of the detached cluster (**Y**_*t*_), number of shearing events (*noe*) and time to a detachment event (*t*). We are interested in developing a surrogate model for predicting the volume of the detached cluster. The input variables for modelling this output are the 7 parameters listed in Table 1 (*μ*_*m*,*HET*_, *K*_*s*,*HET*_, *Y*_*HET*_, *γ*, *K*_*n*_, *γ*_*n*_ and *K*_*e*_) and expected number of shear events (*noe*). We propose to use a dynamic linear model for modelling the log-transformed volume of the detached cluster because of the time series nature of our data. The dynamic linear model is an extension of standard linear regression models with incorporation of time-varying regression coefficients [37]. Therefore, a dynamic estimation of our model parameters will enable us to have a better understanding of the complex problem we are addressing. A dynamic model is usually given as a pair of equations such that for *t* > 0, we have

$$\left\{ \begin{array}{c} Y_{t}=F_{t}\beta_{t}+v_{t},\;v_{t}∼N\left(0,V_{t}\right),\;\left(a\right)\\ \beta_{t}=G_{t}\beta_{t-1}+w_{t},\;w_{t}∼N\left(0,W_{t}\right),\;\left(b\right) \end{array}\right.$$ (1)

where **F**_*t*_ is an *m* × *p* dynamic regression matrix (explanatory variables such that *F*_*t*_ = {*μ*_*m*,*HET*_, *K*_*s*,*HET*_, *Y*_*HET*_, *γ*, *K*_*n*_, *γ*_*n*_, *K*_*e*_, *noe*}) and **G**_*t*_ is an *p* × *p* state evolution matrix. *v*_*t*_ and *w*_*t*_ are two independent Gaussian random vectors with mean 0 and variances **V**_*t*_ and **W**_*t*_, respectively. **W**_*t*_ is the evolution variance matrix for ***β***_*t*_ and **V**_*t*_ is the observation variance matrix while ***β***_*t*_ is an *p* × 1 vector of regression parameters. We assume that matrices of unknown parameters are time-invariant, **G**_*t*_ = **G**, **V**_*t*_ = **V** and **W**_*t*_ = **W**. Suppose further that the matrix of the explanatory variable is also time-invariant, then we have **F**_*t*_ = **F**. Eq 1(a) and 1(b) are usually called observation and state equations, respectively. Let

$$\beta_{0}∼N\left(m_{0},C_{0}\right),$$ (2)

where *m*_0_ and *C*_0_ are known constants fixed in this analysis. Combining these two equations above, we can easily infer that **Y**_*t*_|***β***_*t*_ ∼ *N*(**F**_*t*_ ***β***_*t*_, **V**_*t*_) and ***β***_*t*_|***β***_*t*−1_ ∼ *N*(**G**_*t*_ ***β***_*t*−1_, **W**_*t*_). The two equations above can be applied together for making one-step ahead predictions of our output of interest, ie volume of the detached particle. We can sequentially estimate the dynamic state *β*_*t*_ given the data, using a recursive pair of matrix equations, often referred to as the Kalman filter. For instance, to predict observations **Y**_*t*+1_ based on data **Y**_1:*t*_, we can first estimate *β*_*t*+ 1_ of the state vector and use the estimated values for making predictions **Y**_*t*+1_. In other words, we can obtain the one-step ahead observation predictive density *π*(**Y**_*t*+1_|**Y**_1:*t*_), from one-step ahead state predictive density *π*(**β**_*t*+1_|**Y**_1:*t*_). See Supporting information S1 Text for further details.

Here, we describe a Bayesian method where the unknown parameters are treated as random variables. In our case, the unknown parameters that are required to be estimated are the state evolution matrix **G**_*t*_ and the evolution and observation variance matrices **W**_*t*_ and **V**_*t*_. To simplify our approach and make the problem identifiable, we assume they are diagonal matrices and constant over time. Therefore, we define the matrices of unknown parameters as below

$$\left\{ \begin{array}{c} G_{t}=G=diag\left(\psi_{1},\ldots ,\psi_{k}\right),\\ W_{t}=W=diag\left(\phi_{y,1}^{-1},\ldots ,\phi_{y,m}^{-1}\right),\\ V_{t}=V=diag\left(\phi_{\beta ,1}^{-1},\ldots ,\phi_{\beta ,p}^{-1}\right), \end{array}\right.$$ (3)

and take the parameters of the evolution matrix **G** to be normally independent and identically distributed such that

$$\psi_{j}∼N\left(\psi_{0},\tau_{0}\right),\;j=1,\ldots ,k,$$ (4)

and $\phi_{y,i}^{-1}$ and $\phi_{\beta ,j}^{-1}$ to have independent gamma distributions

$$\left\{ \begin{array}{c} \phi_{y,i}^{-1}=Ga\left(\alpha_{y,i},b_{y,i}\right),\;i=1,\ldots ,m,\\ \phi_{\beta ,j}^{-1}=Ga\left(\alpha_{\beta ,i},b_{\beta ,i}\right),\;j=1,\ldots ,p. \end{array}\right.$$ (5)

Further derivation and a summary of the Gibbs sampling algorithm we use are given in S1 Text of the Supporting information.

### Poisson regression

The Poisson model is employed for modelling event count data, eg the number of shearing events or organisms in an experiment. One of the key properties of count data is that they must be non-negative integers. A Poisson regression model expresses the logarithm of a response or dependent variable (count or rate data) as a linear function of a set of predictor variables. Such a log-linear Poisson model is often adopted to describe a time series of counts or rates. The model assumes the outcome **y**_*k*_ to be Poisson with mean λ_*k*_, so that for a univariate predictor variable *x*_*i*_ (eg biofilm height), the model is

$$y_{k}∼Poisson\left(\lambda_{k}\right),$$ (6)

$$\lambda_{k}\left(x\right)=\text{exp}(\sum_{i=1}^{p}x_{ik}\beta_{i}),$$ (7)

where *k* = 1, …, *n*, λ_*k*_(*x*) is the exponential-mean function and **B** = (***β***_1_,…,***β***_*p*_) is the vector of unknown parameters and x_*ik*_ are the explanatory variables. A discrete random variable **Y** with the probability mass function of **Y** given as

$$f\left(k;\lambda \right)=\text{Pr}\left(Y=k\right)=\frac{\lambda^{k}e^{-\lambda }}{k!},$$ (8)

with parameter λ > 0, for *k* = 0, 1, 2, … is regarded as a Poisson distribution. The mean and variance of a Poisson-distributed random variable are both equal to λ. Parameters **B** are unknown and need to be estimated.

We have seen earlier in Fig 3 (middle column, top-plot) that the number of shear events has different temporal patterns for different shear rates, and also large stochastic variations (third column, top-plot). We apply a Bayesian MCMC algorithm to efficiently estimate our parameters and make reliable predictions, including a measure of uncertainty. Adopting a fully Bayesian approach, the Poisson likelihood function is given by

$$L\left(B\right)=\frac{1}{\prod_{k=1}^{n}y_{i}!}\text{exp}[-\sum_{k=1}^{n}\text{exp}(\sum_{i=1}^{p}x_{ik}\beta_{i})+\sum_{i=1}^{p}\beta_{i}\sum_{k=1}^{n}x_{ik}y_{k}].$$ (9)

Let the prior *π* for parameter ***β***_*i*_ be given as an independent normal distribution with mean *m*_*i*_ and variance *v*_*i*_, ie ***β***_*i*_ ∼ *N*(*m*_*i*_, *v*_*i*_). Under this procedure we will have a joint density of **B** given as

$$\pi \left(\beta_{1},\ldots ,\beta_{p}\right)=\prod_{i=1}^{p}\frac{1}{\sqrt{\left(2\pi v_{i}\right)}}\text{exp}(-\frac{1}{2v_{i}}{\left(\beta_{i}-m_{i}\right)}^{2}).$$ (10)

Using Bayes’ theorem, the posterior distribution is proportional to the product of the likelihood function and the joint prior of all parameters. Here, the posterior distribution of ***β***_*i*_ conditioning on the given data can be obtained by combining Eqs 9 and 10 above as

$$\pi_{y}\left(\beta_{1},\ldots ,\beta_{p}\right)\propto \text{exp}[-\sum_{i=1}^{p}\frac{\beta_{i}^{2}}{2v_{i}}\sum_{i=1}^{p}\alpha_{i}\beta_{i}-\sum_{k=1}^{n}\text{exp}(\sum_{i=1}^{p}x_{ik}\beta_{i})],$$ (11)

where $\alpha_{i}=\frac{m_{i}}{vi}+\sum_{k=1}^{n}x_{ik}y_{k}$. We note that there is no conjugacy between the Poisson likelihood and normal prior distribution which makes exact inference analytically infeasible [19]. We use Poisson regression to model expected number of shear events per unit time and apply Bayesian MCMC to estimate the parameters of the model by assigning a prior distribution on the regression parameters. The expected value for the Poisson model can be derived from the posterior draws of **B** based on the MCMC iterations and is given by

$$E\left(y|X\right)=\lambda_{k}=\text{exp}\left(x_{k}B\right),$$ (12)

while the predictions are draws from the Poisson distribution with parameter λ_*k*_ [38].

## Results

### Procedure for modelling outputs

We use data from the LAMMPS model simulation output. We consider two different simulation datasets in this paper. The first dataset is the expected number of shearing events per unit time. The second dataset is the volume of detached biofilm clusters per unit time. The input variables to the simulator are the seven parameters listed in Table 1. They are *K*_*s*,*HET*_, *μ*_*m*,*HET*_, *Y*_*HET*_, shear rate *γ*, spring coefficient for collision *K*_*n*_, viscous coefficient *γ*_*n*_ and EPS stiffness *K*_*e*_. These seven parameters in addition to the number of shear events *noe* are used for predicting the volume of detached clusters. The four auxiliary variables of total number of particles, EPS composition, biofilm height and mass (Fig 3) are computed summary statistics. These four variables including shear rates and time are used for predicting the expected number of events.

Here, we present the results of our analysis. We based our analysis on the last 200,000 s, corresponding only to the period when the shear flow was applied. We chose to further reduce the dimension of our data by averaging at every 10,000 s which made handling and processing of the data much easier. In other words, our outputs of interest are given as the number of events per 10,000s and detached volume per 10,000s. We have 140 simulations with five replicates for each of them. Our data are averaged and taken to be deterministic. We subdivided our data into two groups. We use 130 data points as a training dataset and use the remaining 10 data points as the test data to verify the performance of our surrogate models.

### Bayesian Poisson results

To proceed with our analyses, we first fitted a Bayesian Poisson regression to the number of shear events as a quadratic function of time, number of particles, shear rates, EPS composition, biofilm height and mass. We used a quadratic model of the form

$$\sum_{i=0}^{p}\sum_{j=0}^{p}\beta_{i,j}x_{ik}x_{jk},$$ (13)

where each **x**_.,*k*_ for *k* = 1, …, *n* represents an explanatory variable (*p* = 6) in this subsection. In the Bayesian context, the data are augmented by a prior distribution. This prior information given over the parameters is then combined with the likelihood function using Bayes theorem to give the posterior distribution of the parameters. We used MCMC to estimate the unknown ***β*** parameters. Our prior for each variable is taken as a normal distribution. To initialize our algorithm we used the maximum likelihood estimates of ***β*** as the starting values. The prior mean *m*_*i*_ is taken as 0.5 for all the six ***β*** values and prior precision $v_{i}^{-1}=0$.

**Fig 4:**
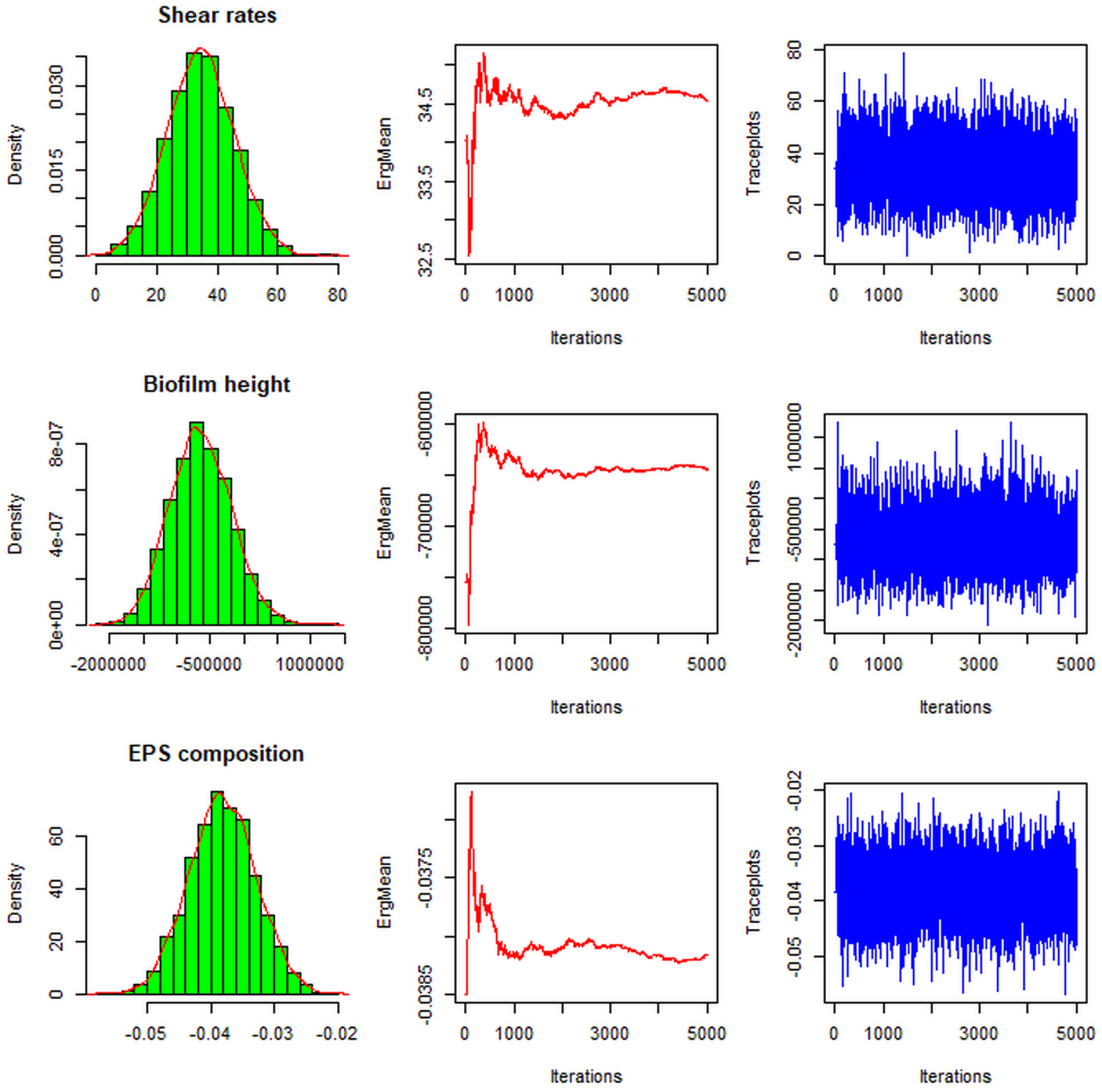
Diagnostic plots showing the convergence of some of the estimated *θ* parameters of Bayesian Poisson model for expected number of events. The first column shows the posterior density of the state variables. The middle column is the running ergodic means of MCMC samples. The third colum is the trace plots for the MCMC samples.

We generate samples from the posterior distribution of Poisson regression given in Eq 11 using a Metropolis algorithm. We run the algorithm for 6,000,000 MCMC iterations, with a burn-in of 1000 samples discarded to remove the influence of the starting point from the estimate. We kept every 1000 iterations (thin = 1000) to reduce the autocorrelation in the saved MCMC samples. Fig 4 shows the diagnostic plots for examination of MCMC samples for convergence. We provide the estimates for the shear rate, biofilm height and EPS composition. The posterior density plots provide information about the shape of posterior distributions of parameters. The ergodic means (middle column) from MCMC samples are relatively stable after 1000 simulations. The ergodic means are computed by a batch mean technique where the stationary Markov chains are divided into different batches after removing the burn-in. The mean and standard error based on the average of the batch means are then calculated. The trace plots indicated a well-mixed chain. We can conclude that the convergence of the MCMC has been reached to estimate the posterior means of unknown parameters. The plots for other parameters are not shown in this paper but also indicated that those parameters have converged.

**Fig 5:**
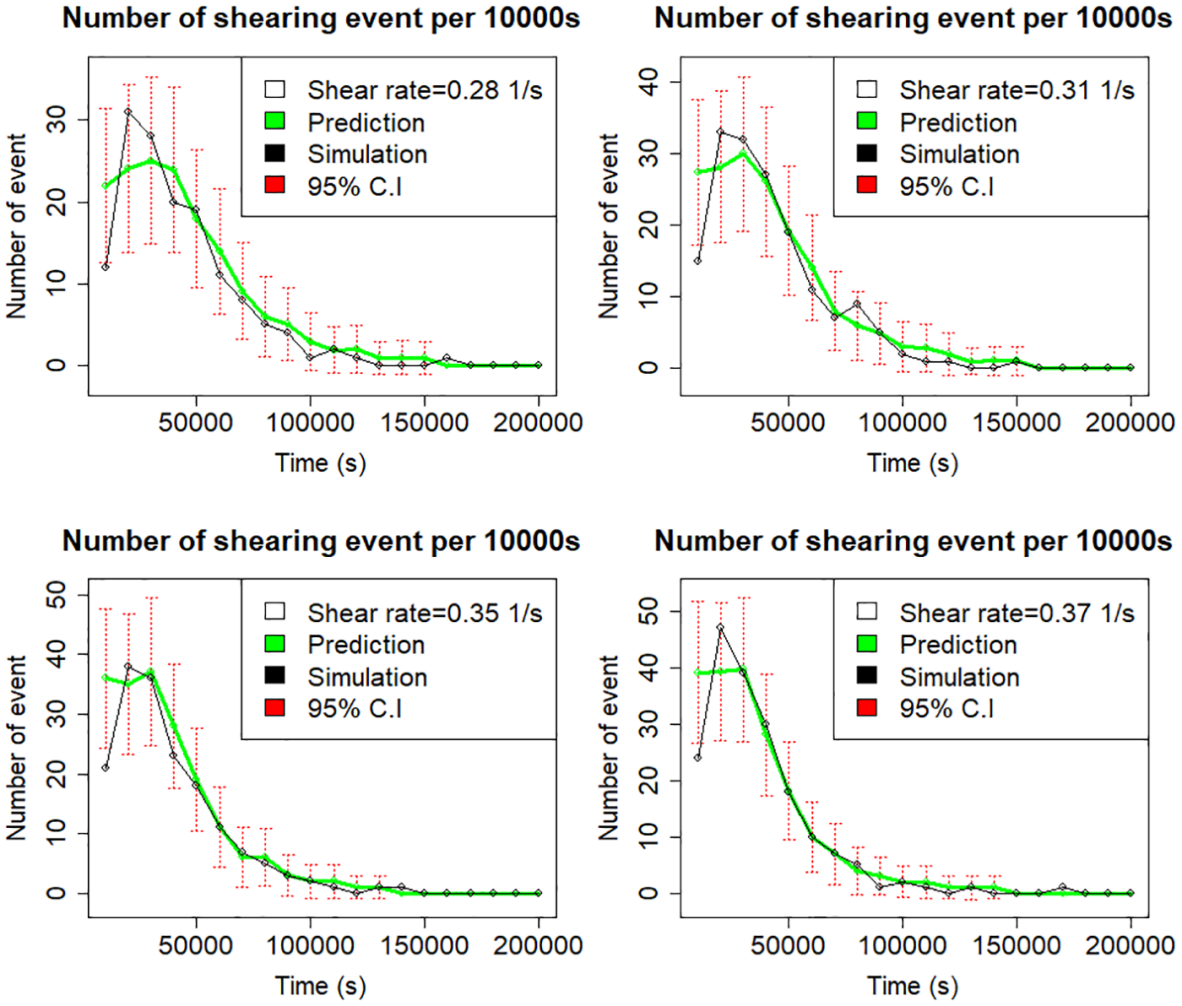
Comparison between the simulation and prediction for expected number of shearing events per 10000s for different shear forces.

Now, we test the performance of fitted Poisson regression models on the 10 left-out observations. Fig 5 is the cross-validation plot that compares the expected number of shear events under four different shear rates from simulation and the predictive model. Each output is plotted against time. As we earlier observed in Fig 2, there is a moderate linear increase in the number of shear events until a threshold value is reached and then gradually declines afterwards. The patterns are consistent with different shear rates. Overall, the four results in this Fig 5 are well predicted as most of the simulated values lie within the 95% probability intervals. We assess the overall performance of Poisson model by computing the root mean squared value (RMSE = 3.24) and percentage of variance explained for the left out data points (*ρ* = 91%).

### Dynamic linear model results

We next fit the dynamic linear model to the volume of detached clusters using Eq 1 where *m* = 130, *p* = 8 here and *k* = *p*. We standardize our input data to range over [0, 1]. This transformation will eliminate the effect of different measurement units and will enable us to get better parameter estimates. The data is normalized by centering the column with their respective minimum values and divided by their range such that *x*′ = (*x* − *x*_*min*_)/(*x*_*max*_ − *x*_*min*_).

We initialized the Markov chain sampler with the following prior hyperparameters; *ψ*_0_ = 0; *τ*_0_ = 1; *α*_**y**,*i*_ = 3; *b*_**y**,*i*_ = 0.01; *α*_***β***,*j*_ = 3; *b*_***β***,*j*_ = 1, *m*_0_ = 0, *C*_0_ = *I*_*p*_, where *I*_*p*_ is a diagonal matrix of ones. We run the DLM algorithm for 1,000,000 MCMC iterations, keeping every 100 iterations (thin = 100) in order to reduce the autocorrelation in the saved MCMC samples where a burn of 5000 samples is removed before making the diagnostic plots.

**Fig 6:**
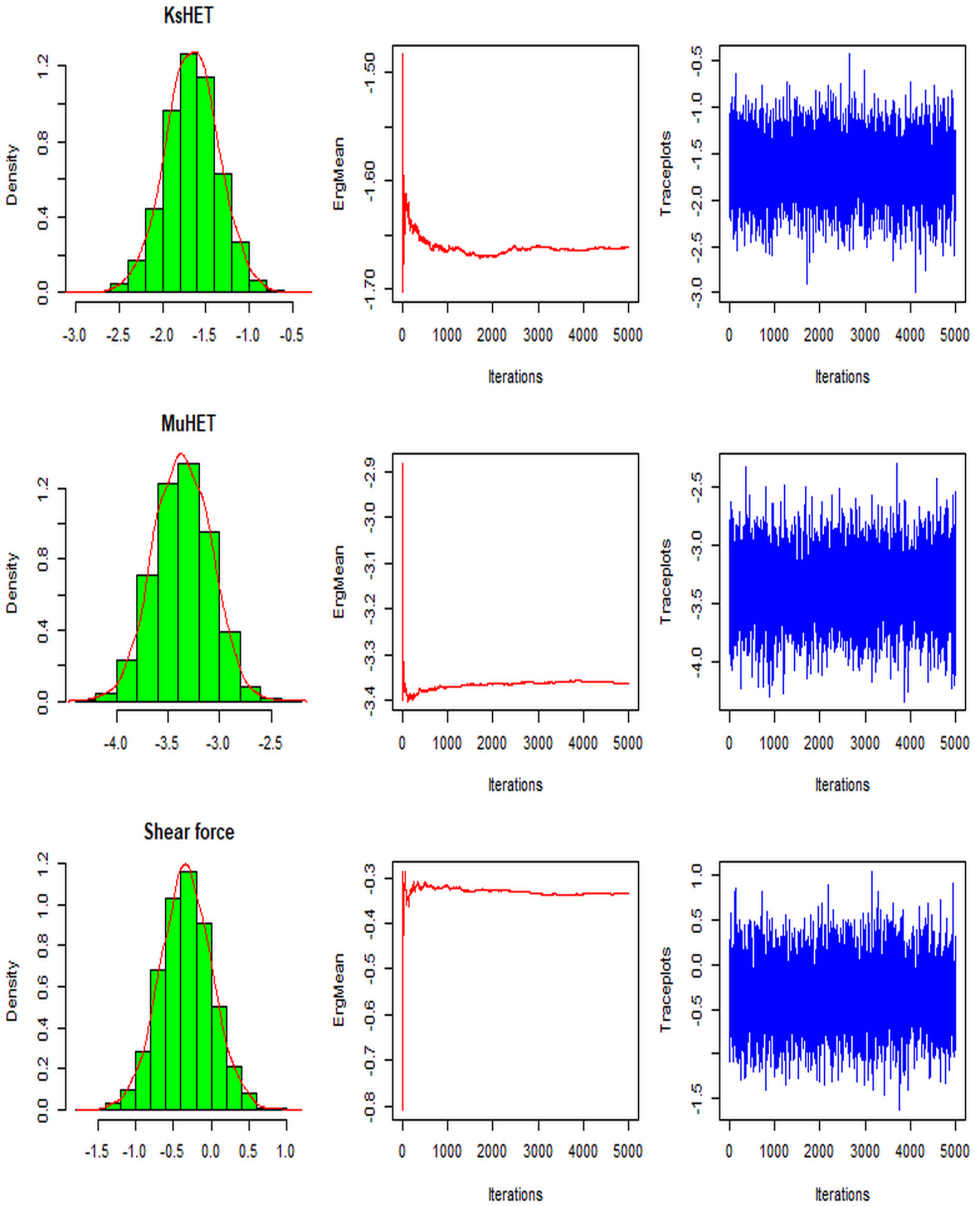
Diagnostics plots showing the convergence of the three randomly chosen regression parameters *θ* of the Bayesian dynamic linear model at time 10000s. The first column shows the posterior density of the state variables. The middle column is the running ergodic means of MCMC samples. The third colum is the trace plots for the MCMC samples.

Fig 6 shows the diagnostic plots obtained from the MCMC outputs for the three randomly selected regression parameters. The posterior density plots provide information about the shape of posterior distributions of parameters. The ergodic means (middle column) from MCMC samples are relatively stable after 1000 iterations. We can conclude that the convergence of the MCMC has been reached to estimate the posterior means. Diagnostic plots for three randomly selected values from the state and observation variance (**V** and **W**) parameters are displayed in the S1 and S2 Figs of the Supporting information. These plots are similar in pattern to Fig 4 in term of convergence while the diagnostic plots for evolution matrix *G* are not shown but also indicated convergence.

Now, we test the performance of fitted models on the left-out observations. S3 Fig of the Supporting information is the cross-validation plot that compares the detached volume under four different shear rates for the simulator and emulator predictions. The plot for each shear rate has a relatively similar pattern. Similar to what we earlier saw in Fig 2 (middle panel), the detached volume grows linearly over time < 40,000*s* for all shear rates and a rapid increase followed by a moderately decreasing trend. There is a consistency in the pattern observed for the four selected shear rates. Overall, the simulated output values and that of predictions are relatively close. The degree of closeness reflects the accuracy of our DLM model. The uncertainty levels are a little bit higher for the first time point compared to the remaining time points. The percentage of variance explained and root mean squared error (RMSE) for this model are 81.5% and 1.023 respectively.

### Sensitivity analysis

To further understand the dynamics of the system we are modelling, the relative contribution of each variable to the total output variance is explored. We perform dynamic sensitivity analysis because of time-dependent nature of our data. We examine how sensitive the log-transformed volume of detached clusters are to changes in parameters over time. We use the Sobol method which calculates indices by variance decomposition. We compute the first order and total indices. Suppose our model is represented by **y**_*t*_ = *f*(**x**_1,*t*_,…,**x**_*p*,*t*_). The first order index is given as $S_{i,t}=\frac{Var[E\left(y|x_{i,t}\right)]}{Var(y_{t})},$ where *Var*[*E*(**y**|*x*_*i*,*t*_)] is partial variance or the main effect of variable *x*_*i*_, and Var(**y**) is the total variance of the response **y** [39].

**Fig 7:**
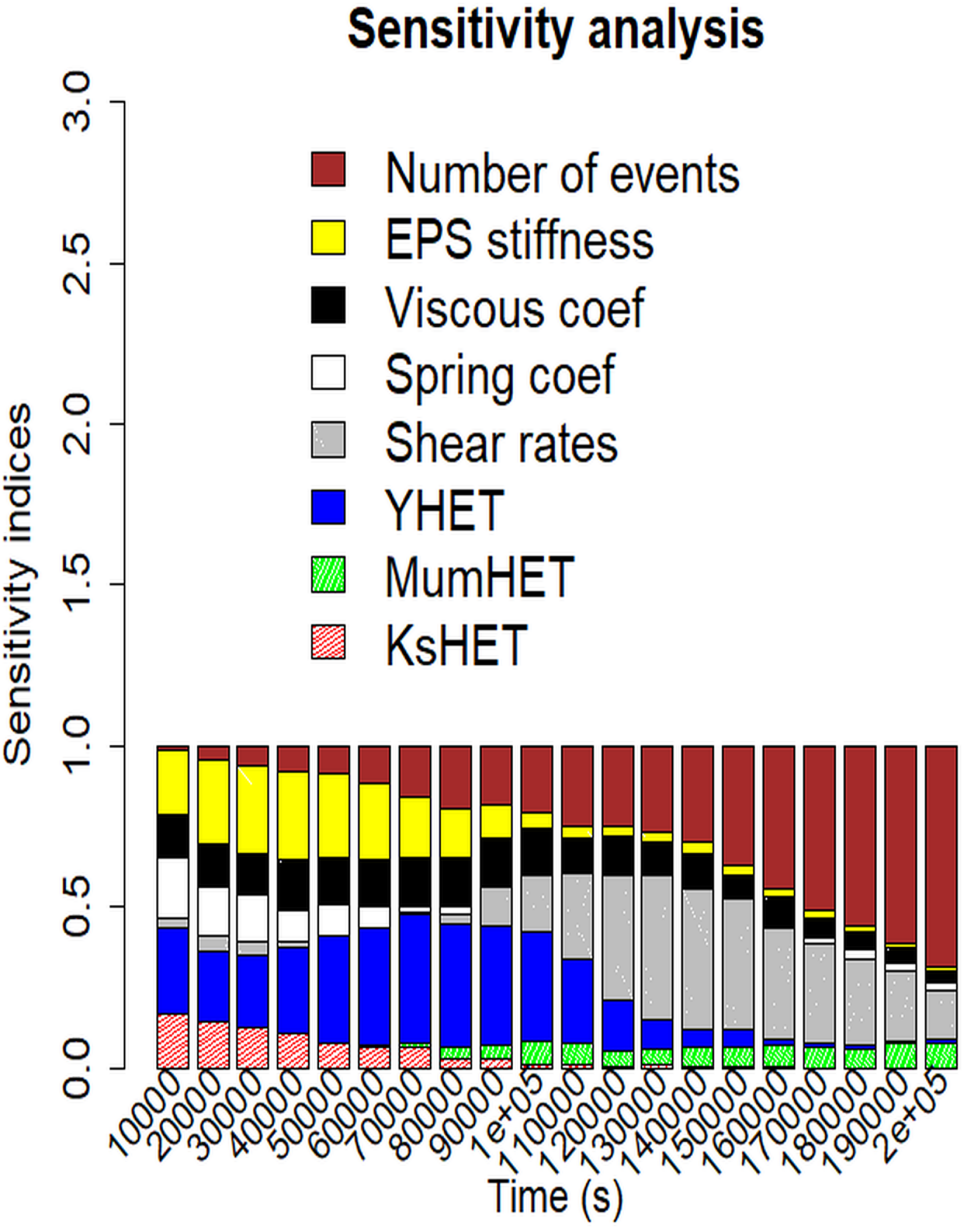
Barplots showing the sensitivity indices of the volume of detached clusters for the eight input variables over time.

We sampled 10,000 observations from a uniform distribution for each of the eight input variables. It is also possible to sample directly from the DLM Markov chain results. The relative importance of each parameter is shown in Fig 7. We observed that detached cluster volume is mostly sensitive to the number of shear events (*noe*), shear rates (*γ*), yield coefficient for heterotrophic bacteria (Y_*HET*_) and EPS stiffness (*K*_*e*_). At earlier times, the sensitivity of EPS stiffness is high and gradually becomes less sensitive at the later time. On the contrary, the sensitivity of the number of shear events grows over time.

While the sensitivity of viscous coefficient is relatively moderate and constant in the first few time points, its index reduces at a later time. The effect of shear rates, on the contrary, is pronounced at later times. The parameters *K*_*s*,*HET*_, *μ*_*HET*_ and spring coefficient have very low indices, an indication that the volume of the detached cluster does not react greatly to a change in these parameter values. It is obvious that sensitivity of the model parameters is temporally dynamic, emphasising the significance of conducting the sensitivity analysis across multiple time points. Overall, the *noe*, *γ*, Y_*HET*_, *K*_*e*_ are the four principal determinants of the volume of detached clusters.

## Discussion and conclusion

There is a significant change in the morphology and dynamics of biofilm formation when a shear flow is applied on a mature biofilm as seen in Fig 1. Also, it is obvious that shear force affects the biofilm structure in line with [2] observations. Moreover, at higher shear rates, a more dense and stable biofilm is likely to be produced because of stronger adherence from EPS matrix than those subjected to lower shear forces.

The role of interactive effects of shear force and other factors like pH and temperature on the biofilm fragmentation should be explored with the surrogate model but we do not currently have access to this simulation data in our study. We also remark that biofilms belong to viscoelastic materials and this property is also a significant determinant of the deformation behaviour of biofilm growing under shear flow as seen in the sensitivity results. In this study, a shear flow is applied to a pre-grown biofilm of certain height to explore the detachment event. It is also possible to simultaneously model both the attachment and detachment, but only the dominant process will be explicitly modelled as noted in [26]. This implies that if the detached velocity is greater the attached velocity only the net detachment will be modelled and there will be no particle attachment.

In summary, the influence of hydrodynamic shear force on biofilm fragmentation has been examined. We have developed a surrogate-based model for quantifying the effect of shear stress on the volume of detached clusters and number of shear events. This paper provides new insights on how advanced statistical techniques can be used to simplify and study biofilm deformation and bacteria detachment. We note that it is essential to develop a cheaper predictive model of biofilm deformation and bacteria detachment in response to mechanical forces and growth parameters because the knowledge can advance the performance and operational stability of wastewater reactors. For instance, the surrogate model can be incorporated into the NUFEB model at mesoscale to produce more refined NUFEB models that are computationally efficient for providing information on a large scale such as WWTP.

The biofilm simulation was initialized and grown for 40000 s without flow then subjected to shear flow to reduce the biofilms size because of the predominance of the breakup process. This results in biofilm of smaller size than the original size due to the shearing event. The volume of biofilm that gets sheared-off and the number of shear event over time are recorded for different shear rates. This study examines the extent to which the shear force affect the number of shear events and volume of detached clusters using a cheaper surrogate model. The joint impact of shear stress and other covariates are examined on biofilm of different sizes. We assume that each occurrence of shearing can be modelled in terms of an event.

We used a 10,000s averaging as a strategy to condense the time series data. It will be interesting to assess the effect of this averaging on our predictive models. In our analyses, we have used normal and gamma priors because they are flexible and widely employed in various applications for modelling with Bayesian MCMC. The limitation of the MCMC algorithm is that the computational cost of a large parameter space is high. We compute the average number of shear events and volume of detached clusters that occur over time. We observe that the number of shear events increases until maximum values after which there is a gradual reduction. We used a Bayesian Poisson log-linear model to relate the expected number of shear events to characterize output summaries from the simulation.

The sensitivity analysis indicated that the number of shear events *noe*, shear rates *γ*, yield coefficient Y_*HET*_ and EPS stiffness *K*_*e*_ are the four primary variables for predicting the volume of detached clusters and are less affected by *K*_*s*,*HET*_ and *μ*_*HET*_. We can conclude that the growth, structure and performance of bacteria biofilms are highly related to the hydrodynamic shear force. The IB model simulation implemented within LAMMPS is computationally expensive, and our surrogate models are much faster to run than the simulator. Under different parameter combinations, it takes an average of between 8-11 hours to simulate both the growth and detachment patterns for about 3 days at 2000s timestep on a Linux cluster machine. Apart from the computational time required to estimate the necessary parameters, the emulator produces the required outputs within ≈ 60*s*.

This approximately 480-fold increase in computational efficiency is particularly useful as a computational tool for the simulation and analysis of multiscale biological systems. This novel combination of advanced statistical techniques for modelling biofilm detachment behaviour using a surrogate-based approach is capable of greatly reducing the computational cost of modelling across large spatial and temporal scales. This study provides a significant step towards improving the performance, robustness and stability of biofilm-based wastewater treatment plant by helping to scale-up agent based models to reactor scale.

## Supporting information

S1 Text Supporting text. Derivation of DLMs, summary of Gibbs sampling algorithm and additional Table referenced in the original article.
(PDF)

S1 Fig Diagnostic plots. Plots showing the convergence of the three *V*_1_, *V*_2_ and *V*_3_ randomly chosen observation variance parameters of the Bayesian dynamic linear model. The first column shows the posterior density of the observation variances. The middle column is the running ergodic means of MCMC samples. The third colum is the traceplots for the MCMC samples.
(TIF)

S2 Fig Diagnostic plots. Plots showing the convergence of the three randomly chosen *W*_1_, *W*_2_ and *W*_3_ state variance parameters of the Bayesian dynamic linear model. The first column shows the posterior density of the state variances. The middle column is the running ergodic means of MCMC samples. The third colum is the trace plots for the MCMC samples.
(TIF)

S3 Fig Model comparison. Comparison between the simulation and prediction for log-transformed detached volume over time for different shear forces. The results are normalized by initial biofilm volume.
(TIF)

S4 Fig Time series plots. Expected number of shear events and volume of detached clusters for different spring coefficients for elastic collision.
(TIF)
