# Supplementary material for: A Bayesian approach to modelling the impact of hydrodynamic shear stress on biofilm deformation
Source: PLoS One. 2018 Apr 12;13(4):e0195484. doi: 10.1371/journal.pone.0195484 (PMC5896950; doi:10.1371/journal.pone.0195484)
Supplement: S1 Text — Derivation of DLMs, summary of Gibbs sampling algorithm and additional Table referenced in the original article. (PDF) [file pone.0195484.s001.pdf]

Supporting information: "A Bayesian approach to modelling the impact of hydrodynamic shear stress on biofilm deformation"

Oluwole K. Oyebamiji[1*], Darren J. Wilkinson[1], Pahala Gedara Jayathilake[2], Steve P. Rushton[3], Ben Bridgens[2], Bowen Li[4], Paolo Zuliani[4]

1 School of Mathematics, Statistics and Physics, Newcastle University, Newcastle upon Tyne, NE1 7RU, U.K.

2 School of Engineering, Newcastle University, Newcastle upon Tyne, NE1 7RU, U.K.

3 School of Natural and Environmental Sciences, Newcastle University, Newcastle upon Tyne, NE1 7RU, U.K.

4 School of Computing Science, Newcastle University, Newcastle upon Tyne, NE4 5TG, U.K.

*Corresponding author: wolemi2@yahoo.com

Derivation of DLMs

Let $\boldsymbol{\beta}_t$ be a Markov chain under some regularity conditions, and suppose that each $\mathbf{Y}_t'^s$ are independent conditionally on $\boldsymbol{\beta}_t$, if we also assume further that $\mathbf{Y}_t$ depends only on $\boldsymbol{\beta}_t$. Therefore, equation A.1 follows directly from equations **1** and **2** in the original article which completely determines any given state space model, and other distributions can be easily derived using this equation

$$\pi(\boldsymbol{\beta}_{0:t}, \mathbf{y}_{1:t}) = \pi(\boldsymbol{\beta}_0). \prod_{j=1}^{t} \pi(\boldsymbol{\beta}_j|\boldsymbol{\beta}_{j-1})\pi(\mathbf{y}_j|\boldsymbol{\beta}_j), \qquad t > 0 \tag{A.1}$$

where $\pi(\boldsymbol{\beta}_0)$ is the initial distribution and $\pi(\boldsymbol{\beta}_t|\boldsymbol{\beta}_{t-1})$ and $\pi(\mathbf{y}_t|\boldsymbol{\beta}_t)$ are conditional densities; see details in [1] and [2]. For a general state space model, we use the relations

given in equation A.2 below

$$\begin{cases} \pi(\boldsymbol{\beta}_t|\mathbf{y}_{1:t-1}) = \int \pi(\boldsymbol{\beta}_t|\boldsymbol{\beta}_{t-1})\pi(\boldsymbol{\beta}_{t-1}|\mathbf{y}_{1:t-1})d\boldsymbol{\beta}_{t-1} & \text{state predictive density} \\ \pi(\mathbf{y}_t|\mathbf{y}_{1:t-1}) = \int \pi(\mathbf{y}_t|\boldsymbol{\beta}_t)\pi(\boldsymbol{\beta}_t|\mathbf{y}_{1:t-1})d\boldsymbol{\beta}_t & \text{observation predictive density} \\ \pi(\boldsymbol{\beta}_t|\mathbf{y}_{1:t}) = \dfrac{\pi(\mathbf{y}_t|\boldsymbol{\beta}_t)\pi(\boldsymbol{\beta}_t|\mathbf{y}_{1:t-1})}{\pi(\mathbf{y}_t|\mathbf{y}_{1:t-1})} & \text{filtering density,} \end{cases} \tag{A.2}$$

and suppose further that the random vectors $(\boldsymbol{\beta}_0, \ldots, \boldsymbol{\beta}_t, \mathbf{Y}_1, \ldots, \mathbf{Y}_t)$ are normally distributed, and for any time $t > 0$, then the marginal and conditional distributions are also normally distributed which are completely specified by their means and variances. Therefore, using a normality assumption in conjunction with dynamic linear models defined in equations **2** and A.2, and suppose that $\boldsymbol{\beta}_t|\mathbf{y}_{1:t-1} \sim N(m_{t-1}, C_{t-1})$, we can deduce easily that the one-step ahead predictive distributions for $\boldsymbol{\beta}_t|\mathbf{y}_{1:t-1}$, $\mathbf{Y}_t|\mathbf{y}_{1:t-1}$ and filtering distribution of $\boldsymbol{\beta}_t|\mathbf{y}_{1:t}$ are given respectively as

$$\begin{cases} \boldsymbol{\beta}_t|\mathbf{y}_{1:t-1} \sim N(a_t, \mathbf{R}_t), \quad \text{where} \ \ a_t = \mathbf{G}_t m_{t-1}, \quad \mathbf{R}_t = \mathbf{G}_t\mathbf{C}_{t-1}\mathbf{G}_t' + \mathbf{W}_t, \\ \mathbf{y}_t|\mathbf{y}_{1:t-1} \sim N(f_t, \mathbf{Q}_t), \quad \text{where} \ \ f_t = \mathbf{F}_t a_t, \quad \mathbf{Q}_t = \mathbf{F}_t\mathbf{R}_t\mathbf{F}_t' + \mathbf{V}_t, \\ \boldsymbol{\beta}_t|\mathbf{y}_{1:t} \sim N(m_t, \mathbf{C}_t), \quad \text{where} \ \ m_t = a_t + \mathbf{A}e_t, \quad \mathbf{C}_t = \mathbf{R}_t + \mathbf{A}\mathbf{F}_t\mathbf{R}_t + \mathbf{W}_t, \end{cases} \tag{A.3}$$

where $e_t = \mathbf{Y}_t - f_t$ and $\mathbf{A} = \mathbf{R}_t + \mathbf{F}_t'\mathbf{Q}_t^{-1}$. We already know that the joint distribution of states and observations is Gaussian, when all the parameters of a dynamic regression are known it is relatively easy to derive conditional distributions of states or future observations conditional on the given data. These parameters are unknown, and a major problem in time series analysis is their estimation. One popular approach is to use the maximum likelihood estimation (MLE) framework by maximising the likelihood function for the statistical parameter estimation. The limitations of MLE are the presence of many local maximal and flat likelihood and failure to always account for the uncertainty associated with the estimation of such parameters.

Follow from the main paper and given the observations $\mathbf{y}_{1:T}$, we can obtain the joint posterior density of the states $\boldsymbol{\beta}_{0:T}$ and unknown parameters $\boldsymbol{\Psi} = (\psi_j, \phi_{\mathbf{y},i}^{-1}, \phi_{\boldsymbol{\beta},j}^{-1})$ which is proportional to their joint density such that

$$\begin{cases}\pi(\mathbf{y}_{1:T}, \boldsymbol{\beta}_{0:T}, \boldsymbol{\Psi}) = \prod_{t=1}^{T} \pi(\mathbf{y}_t|\boldsymbol{\beta}_t, \phi_{\mathbf{y}}). \prod_{t=1}^{T} \pi(\boldsymbol{\beta}_t|\boldsymbol{\beta}_{t-1}, \\ \phi_{\boldsymbol{\beta},1}, \ldots, \phi_{\boldsymbol{\beta},p})\pi(\boldsymbol{\beta}_0) \prod_{i=1}^{m} \pi(\phi_{\mathbf{y},i}) \prod_{j=1}^{p} \pi(\phi_{\boldsymbol{\beta},i}).\end{cases} \tag{A.4}$$

It is always difficult to obtain the posterior distribution defined above in a closed form and as a result we can result to numerical approximation using MCMC methods. In particular, the joint posterior of the states and model parameters can be approximated by Gibbs sampling. The full conditionals of $\phi_{\mathbf{y},i}^{-1}$ and $\phi_{\boldsymbol{\beta},j}^{-1}$ can be derived from their joint density and are both Gamma distributions given below as

$$\begin{cases}\phi_{\mathbf{y},i}|\ldots \sim Ga\left(\alpha_{\mathbf{y},i} + \frac{T}{2}, b_{\mathbf{y},i} + \frac{1}{2}S_{\mathbf{y},i}\right), & i = 1, \ldots, m, \\ \phi_{\boldsymbol{\beta},j}|\ldots \sim Ga\left(\alpha_{\boldsymbol{\beta},j} + \frac{T}{2}, b_{\boldsymbol{\beta},j} + \frac{1}{2}S_{\boldsymbol{\beta},j}\right), & j = 1, \ldots, p,\end{cases} \tag{A.5}$$

where $S_{\mathbf{y},i} = \sum_{t=1}^{T}(\mathbf{y}_{i,t} - (\mathbf{F}\boldsymbol{\beta}_t)i)^2$ and $S_{\boldsymbol{\beta},j} = \sum_{t=1}^{T}(\boldsymbol{\beta}_{j,t} - (\mathbf{G}\boldsymbol{\beta}_{t-1})_j)^2$.

Therefore, the sampler can be run to draw a sample from both the full conditional distributions of the states $(\phi_{\mathbf{y},i}|\ldots)$ and $(\phi_{\boldsymbol{\beta},j}|\ldots)$. The Gibbs sampler is an efficient technique for approximating the joint distribution. The mechanism of Gibbs sampling involves iteratively simulating from full conditional distributions. Lastly, the full conditional of ψ_j is derived by combining the prior distribution with the theory of normal distributions such that

$$\psi_j|\ldots \sim N(\psi_{j,T}, \tau_{j,T}), \qquad j = 1, \ldots, k, \tag{A.6}$$

with $\psi_{j,T} = \tau_{j,T}\left[\phi_{\boldsymbol{\beta},j}\sum_{t=1}^{T}\boldsymbol{\beta}_{j,t-1}\boldsymbol{\beta}_{j,t} + \frac{1}{\tau_{j,0}\psi_0}\right]$ and $\tau_{j,T} = \left[\frac{1}{\tau_0} + \phi_{\boldsymbol{\beta},j}\sum_{t=1}^{T}\boldsymbol{\beta}_{j,t-1}^2\right]^{-1}$. See [3, 4, 5] for further background on Gibbs sampling. The summary of this algorithm is given below.

Summary of Gibbs sampling algorithm

Derive the posterior conditionals for each of the random variables in the model from the full density.

Simulate samples from the posterior joint distribution based on the posterior

conditionals.

- Initialize: set $\phi_{\mathbf{y}} = \phi_{\mathbf{y}}^{(0)}$; $\phi_{\boldsymbol{\beta}} = \phi_{\boldsymbol{\beta}}^{(0)}$; $\psi = \psi^{(0)}$ For $k = 1, \ldots, N$:
- Draw $\boldsymbol{\beta}_{0:T}^{(k)}$ from $\pi(\boldsymbol{\beta}_{0:T}|\mathbf{y}_T, \phi_{\mathbf{y}} = \phi_{\mathbf{y}}^{(k-1)}, \phi_{\boldsymbol{\beta}} = \phi_{\boldsymbol{\beta}}^{(k-1)}, \psi = \psi^{(k-1)})$ with FFBS
- Draw $\phi_{\mathbf{y}}^{(k)}$ from $\pi(\phi_{\mathbf{y}}|\mathbf{y}_T, \boldsymbol{\beta}_{0:T} = \boldsymbol{\beta}_{0:T}^{(k)}, \phi_{\boldsymbol{\beta}} = \phi_{\boldsymbol{\beta}}^{(k-1)}, \psi = \psi^{(k-1)})$
- Draw $\phi_{\boldsymbol{\beta}}^{(k)}$ from $\pi(\phi_{\boldsymbol{\beta}}|\mathbf{y}_T, \boldsymbol{\beta}_{0:T} = \boldsymbol{\beta}_{0:T}^{(k)}, \phi_{\mathbf{y}} = \phi_{\mathbf{y}}^{(k)}, \psi = \psi^{(k-1)})$
- Draw $\psi^{(k)}$ from $\pi(\psi|\mathbf{y}_T, \boldsymbol{\beta}_{0:T} = \boldsymbol{\beta}_{0:T}^{(k)}, \phi_{\mathbf{y}} = \phi_{\mathbf{y}}^{(k)}, \phi_{\boldsymbol{\beta}} = \phi_{\boldsymbol{\beta}}^{(k)})$

Table A: List of parameters that remain unchanged for all simulation experiments

Index	Parameters	Symbol	Values	Units	References
1	Decay coefficient	b	0.0133	h^{-1}	[6]
2	EPS formation coefficient	YEPS	0.18	$gCOD/gN$	[7]
3	Diffusion coefficient for substrate	D_s	0.0000000016	m^2s^{-1}	[8]
4	Bulk substrate concentration	Sub	0.00010	kgCOD m^{-3}	Chosen
5	Dynamic viscosity	μ	1×10^{-3}	Pas	(For water)

References

1. Petris G, Petrone S, Campagnoli P. Dynamic linear models. In: Dynamic Linear Models with R. Springer; 2009. p. 31–84.
2. Petris G, Petrone S, et al. State space models in R. Journal of Statistical Software. 2011;41(4):1–25.
3. Harrison J, West M. Bayesian forecasting & dynamic models. Springer New York; 1999.
4. Doss H, Narasimhan B. Bayesian Poisson regression using the Gibbs sampler: Sensitivity analysis through dynamic graphics. Technical report, Citeseer; 1994.
5. Casella G, George EI. Explaining the Gibbs sampler. The American Statistician. 1992;46(3):167–174.
6. Rittmann BE, Schwarz A, Eberl H, Morgenroth E, Perez J, van Loosdrecht M, et al. Results from the multi-species Benchmark Problem (BM3) using one-dimensional models. Water Science and Technology. 2004;49(11-12):163–168.

7. Ni BJ, Fang F, Xie WM, Sun M, Sheng GP, Li WH, et al. Characterization of extracellular polymeric substances produced by mixed microorganisms in activated sludge with gel-permeating chromatography, excitation–emission matrix fluorescence spectroscopy measurement and kinetic modeling. Water Research. 2009;43(5):1350–1358.

8. Alpkvist E, Picioreanu C, van Loosdrecht M, Heyden A. Three-dimensional biofilm model with individual cells and continuum EPS matrix. Biotechnology and bioengineering. 2006;94(5):961–979.
