# Supplementary figures and images for: A Bayesian approach to modelling the impact of hydrodynamic shear stress on biofilm deformation
Source: PLoS One. 2018 Apr 12;13(4):e0195484. doi: 10.1371/journal.pone.0195484 (PMC5896950; doi:10.1371/journal.pone.0195484)

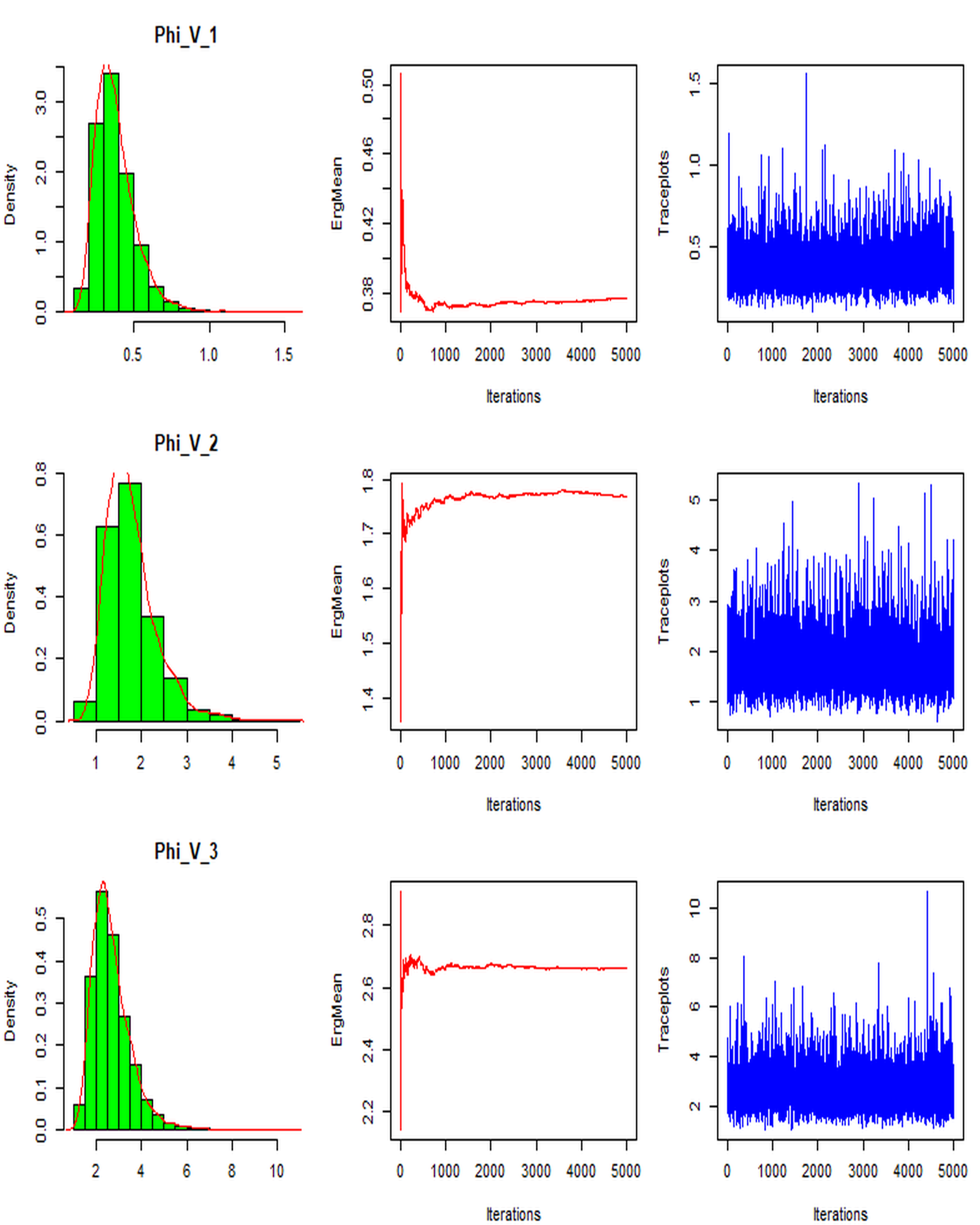

Supplement: S1 Fig — Plots showing the convergence of the three V1, V2 and V3 randomly chosen observation variance parameters of the Bayesian dynamic linear model. The first column shows the posterior density of the observation variances. The middle column is the running ergodic means of MCMC samples. The third colum is the traceplots for the MCMC samples. (TIF) [file pone.0195484.s002.tif]

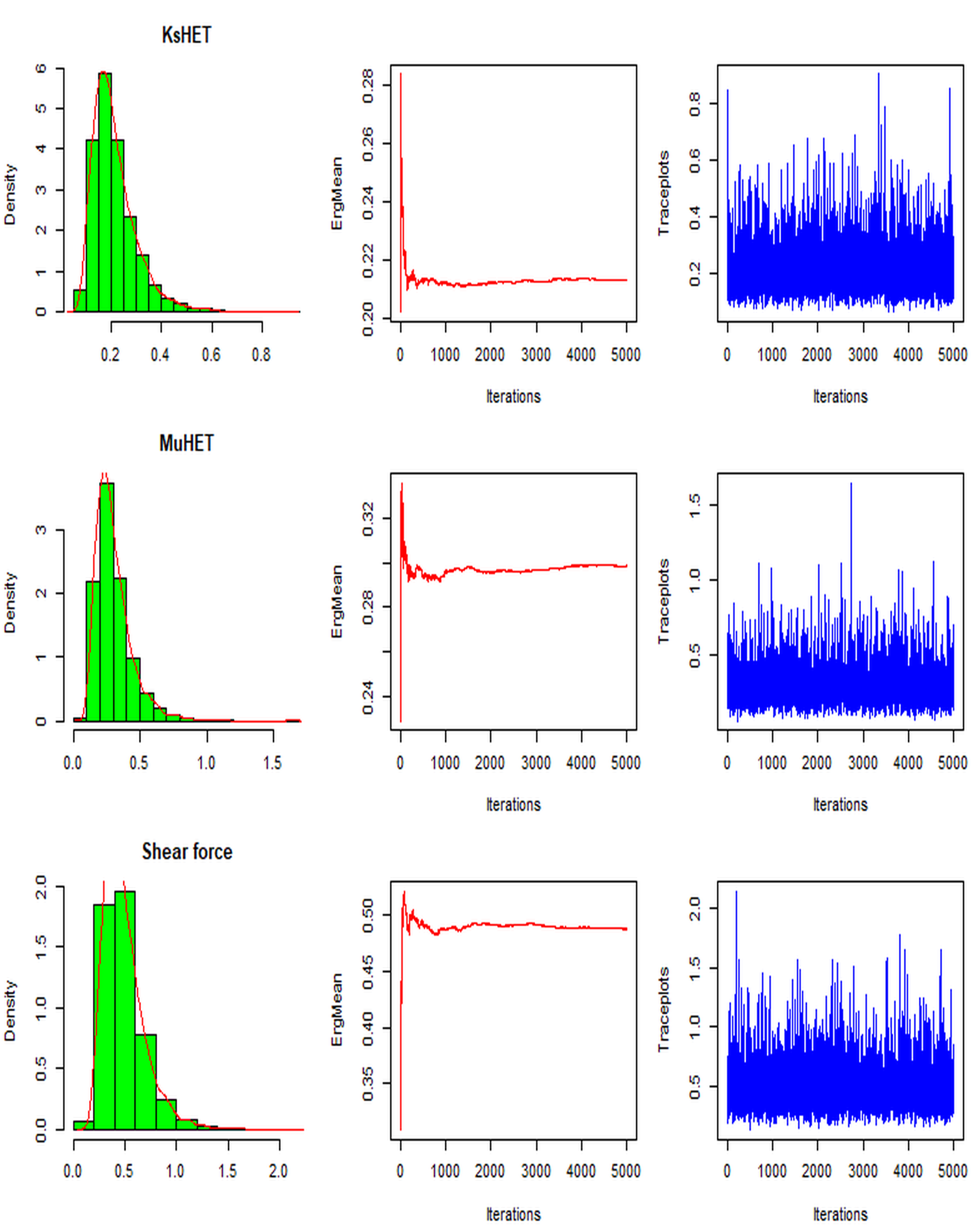

Supplement: S2 Fig — Plots showing the convergence of the three randomly chosen W1, W2 and W3 state variance parameters of the Bayesian dynamic linear model. The first column shows the posterior density of the state variances. The middle column is the running ergodic means of MCMC samples. The third colum is the trace plots for the MCMC samples. (TIF) [file pone.0195484.s003.tif]

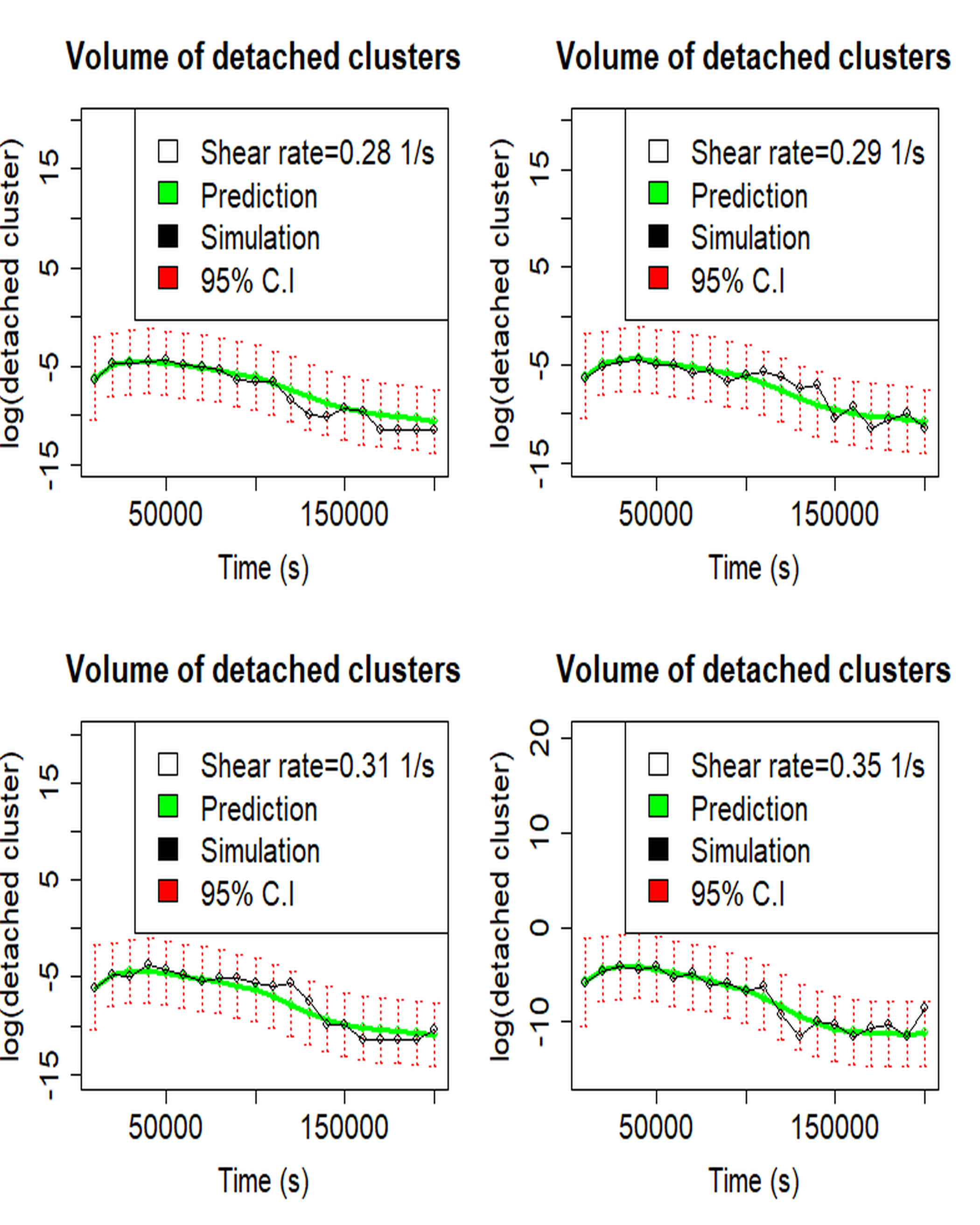

Supplement: S3 Fig — Comparison between the simulation and prediction for log-transformed detached volume over time for different shear forces. The results are normalized by initial biofilm volume. (TIF) [file pone.0195484.s004.tif]

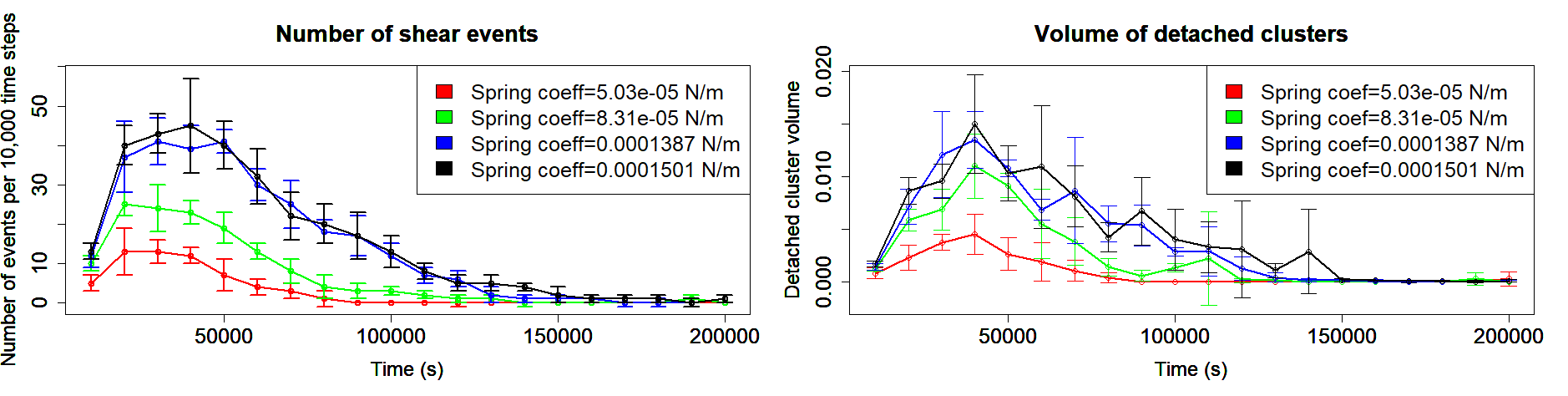

Supplement: S4 Fig — Expected number of shear events and volume of detached clusters for different spring coefficients for elastic collision. (TIF) [file pone.0195484.s005.tif]
